# Effect of different loads on the shoulder in abduction postures: a finite element analysis

**DOI:** 10.1038/s41598-023-36049-9

**Published:** 2023-06-11

**Authors:** Zhengzhong Yang, Guangming Xu, Jiyong Yang, Zhifei Li

**Affiliations:** 1 Shenzhen Pingle Orthopedic Hospital & Shenzhen Pingshan Traditional Chinese Medicine Hospital, affiliate Guangzhou University of Chinese Medicine, No. 15 Lanjin Road, Pingshan District, Shenzhen, 518118 Guangdong China; 2 Department of Orthopaedics, Shenzhen Hospital of Integrated Traditional Chinese and Western Medicine, Guangzhou University of Chinese Medicine, Baoan District, No. 3 Shajin Road, Shenzhen, 518104 Guangdong China; 3 Guangzhou University of Chinese Medicine, No. 232 Waihuan Road, Panyu District, Guangzhou, 510000 Guangdong China; 4 Department of Spine Surgery, The First Affiliated Hospital of Guangxi University of Chinese Medicine, Nanning, 530023 Guangxi China

**Keywords:** Health care, Medical research

## Abstract

Load can change the mechanical environment of dynamic and static stable structures of the shoulder joint, increase the risk of tissue damage and affect the stability of the shoulder joint, but its biomechanical mechanism is still unclear. Therefore, a finite element model of the shoulder joint was constructed to analyze the mechanical index changes of shoulder joint abduction under different loads. The stress of the articular side on the supraspinatus tendon was higher than that of the capsular side, with a maximum difference of 43% due to the increased load. For the deltoid muscle and glenohumeral ligaments, increases in stress and strain were obvious in the middle and posterior deltoid muscles and inferior glenohumeral ligaments. The above results indicate that load increases the stress difference between the articular side and the capsular side on the supraspinatus tendon and increases the mechanical indices of the middle and posterior deltoid muscles, as well as the inferior glenohumeral ligament. The increased stress and strain in these specific sites can lead to tissue injury and affect the stability of the shoulder joint.

## Introduction

Upper limb load bearing training is a common exercise and rehabilitation method for shoulder muscles that can effectively improve the muscle quality^1–4^. To improve or restore the quality of the deltoid muscle, the most common exercise is upper limb abduction training, such as in patients with deltoid atrophy and those performing fitness exercises^5,6^. However, abduction mainly depends on the synergistic effect of the supraspinatus and deltoid muscles^7,8^. Excessive or heavy exercise can easily cause chronic shoulder muscle injury and humeral head movement. In severe cases, it can cause shoulder instability and rotator cuff injury^9–11^. Therefore, it is particularly important to understand the internal mechanical factor of shoulder abduction. At present, experimental biomechanical methods are commonly used to study the in vivo mechanics of shoulder joints. Christopher et al.^12^ used a dynamic shoulder simulator to test the maximum abduction force of the deltoid muscle during the abduction process. Danil et al.^13^ analyzed the stress of the shoulder joint in normal patients with rotator cuff tears during abduction through biomechanical experiments. However, due to the limitations of the experimental environment and conditions, the results of many indicators are difficult to predict.

The finite element analysis method can simulate working conditions that are difficult to carry out in biomechanics and obtain the corresponding mechanical results; it has been widely used in research on the shoulder joints. Filardi et al.^14^ used shoulder finite element analysis to analyze the stress distribution of the humerus during upper arm elevation and abduction. Sabesan et al.^15^ constructed a 3D finite element model to compare the biomechanical differences between two enhanced glenoid designs. The three-dimensional finite element model constructed by Matthew et al.^16^ revealed the stress distribution of the humerus and cartilage under a shoulder load. However, the above shoulder model has different degrees of simplification, such as incomplete construction of the anterior, middle, and posterior deltoid bundle, joint capsule, ligament and other soft tissues, which fails to simulate biological mimicry realistically, affecting the accuracy of the simulation results. At present, the biomechanical effect of load bearing on shoulder tissue is still unclear.

Therefore, this paper aimed to establish a full finite element model of shoulders to analyze the mechanical response of shoulder joints under loading conditions. A model containing component such as the clavicle, scapula, humerus, deltoid muscle, rotator cuff, coraco-clavicular ligament (CCL), acromio-clavicular ligament (ACL), glenohumeral ligaments (GHL), including: superior glenohumeral ligament (SGHL), middle glenohumeral ligament (MGHL), inferior glenohumeral ligament (IGHL) and joint capsule (JC) was developed. To simulate the mechanical effects of different loading states on the shoulder abduction motion, we analyzed the mechanical change mechanism of shoulder abduction and its influence on shoulder tissue.

## Materials and methods

### Computational model

A 30-year-old male volunteer with a height of 169 cm and weight of 72 kg was selected. The volunteer had no history of shoulder injury or disease, such as shoulder pain, shoulder dislocation or fracture, and no abnormalities on imaging examination. The experiment was carried out in accordance with the Code of Ethics of the World Medical Association (Declaration of Helsinki) and approved by the Ethics Committee of the Affiliated Hospital of Guangzhou University of Traditional Chinese Medicine (KY2022088), and the volunteer signed an informed consent form.

**Figure 1:**
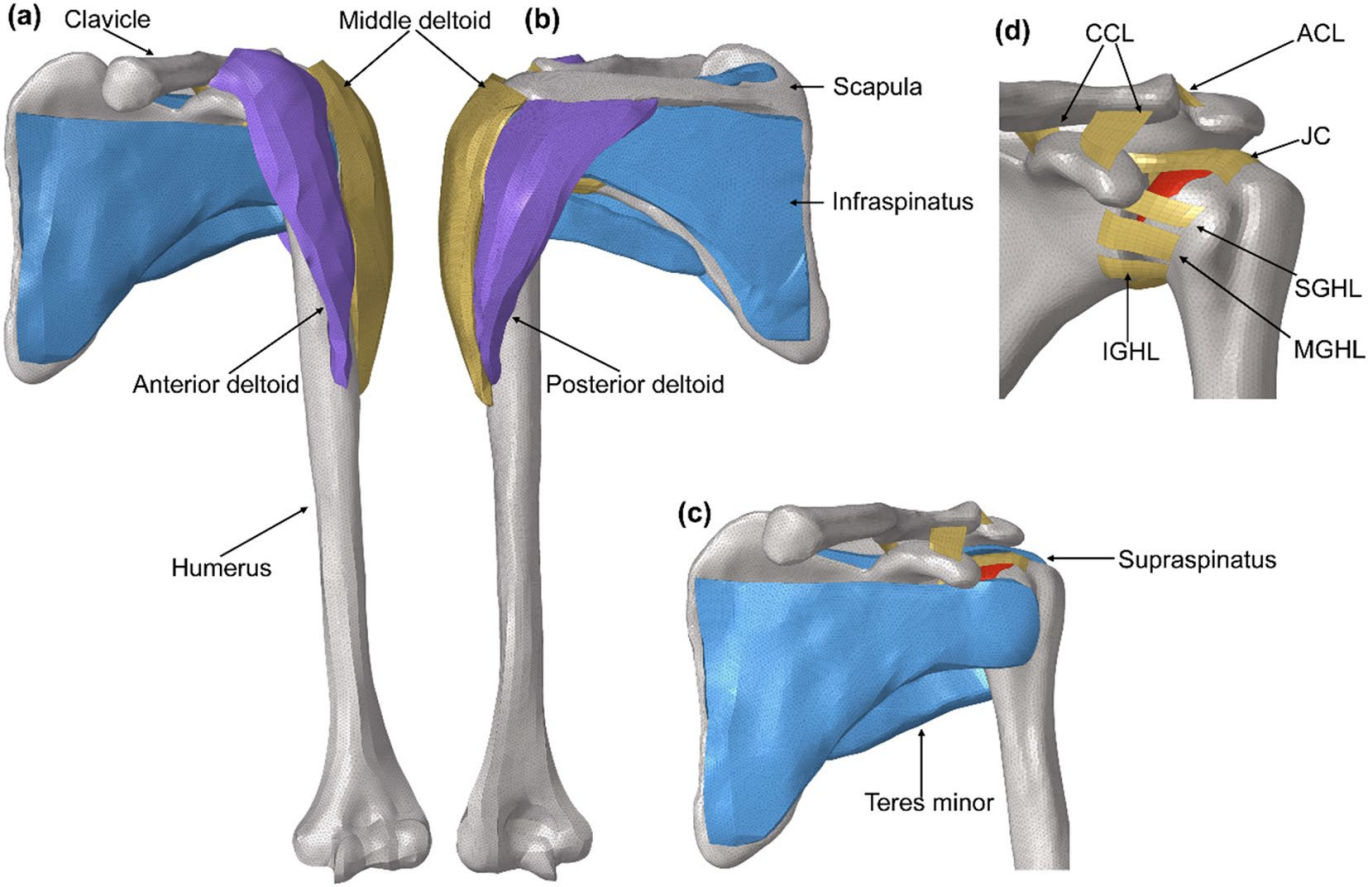
3D finite element model of shoulder joint. (**a**) Anterior view of finite element models of shoulder joint. (**b**) Rear view of finite element models of shoulder joint. (**c**) Shoulder model after removed deltoid muscle. (**d**) Distribution of the shoulder ligament and joint capsule.

To construct the bone and muscle tissue of the three-dimensional finite element model of the shoulder joint complex, CT and MRI examinations of the left shoulder joint were performed, and the files were saved in DICOM format. CT images were imported into Mimics, and the geometric quality models of the clavicle, scapula and humerus were obtained by image processing and optimization, which were saved and exported. The file was imported into Geomagic software, where it was repaired, smoothed, surfaces were built and other processing was performed, and then it was imported into solidwork to generate the entity. The rotator cuff and deltoid muscle were constructed based on MRI images, and the joint capsule and ligament were 2D structures constructed by anatomical position. Finally, the file was imported into a hypermesh, the mesh was divided, and the boundary was set. The total number of elements in the model was 482,594. The shoulder joint model is shown in Fig. 1, and the selection of element types is shown in Table 1^17–19^.

**Table1:** Material property table of the components.

Component	Young's modulus (MPa)	Poisson’s ratio
Cortical bone		
Humerus	15,000	0.3
Scapula	15,000	0.3
Clavicle	17,000	0.3
Cancellous bone	1000	0.3
Articular Cartilage	15	0.45
Muscle		
Supraspinatus	Nonlinear	
Infraspinatus	Nonlinear	
Teres minor	Nonlinear	
Subscapularis	Nonlinear	
Deltoid	Nonlinear	
Ligament		
ACL	10.4	0.3
CCL	9.6	0.3
GHL	150	0.3

### Model validation

1. Verification of the anatomical data.

We measured the morphology of the clavicle, humerus and scapula and compared them with those reported in the previous literature^20–22^.

2. Supraspinatus traction verification.

According to the mechanical loading method in the literature^23^, the mechanical loading conditions of the supraspinatus muscle pulling experiment were simulated, and the strain of the supraspinatus muscle was analyzed and compared with the experimental data.

### Mechanical influence of boundary conditions and different loads on the shoulder joint

The boundary conditions and mechanical loading conditions of this study were based on previous studies^19,24^, which 96N and 144N were applied to the supraspinatus and deltoid muscle around the junction of the humerus, respectively. The scapula and clavicle were fixed, and loads of 0 kg (Group 1), 2.5 kg (Group 2), and 5 kg (Group 3) were given to the distal humerus^25^ to simulate different load states. The stress of the supraspinatus muscle bursa side and joint side, the stress changes of the anterior, middle and posterior deltoid bundle, and the traction strain changes of the glenohumeral ligament were analyzed to evaluate the influence of loading on the shoulder joint abductor muscle and its stability.

## Results

### Verification of the anatomical data

**Figure 2:**
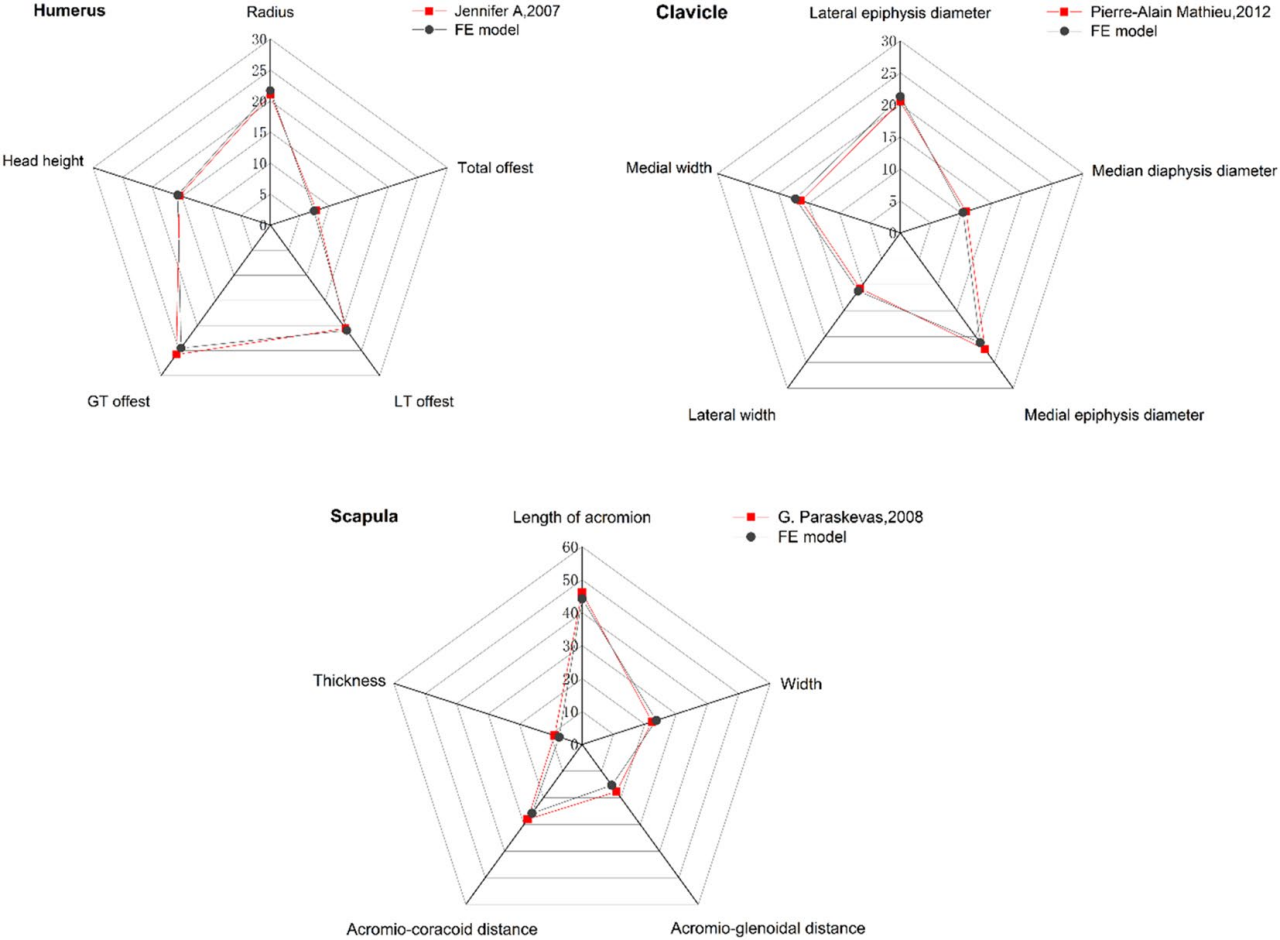
Anatomical verification of clavicle, scapula and humerus.

The morphology and normal angle of the clavicle, scapula and proximal humerus (Lesser tuberosity anterior offset (LT offest), Greater tuberosity lateral offset (GT offest) were measured. The results are shown in Fig. 2, which are all within the range found in the literature^20–22^.

### Supraspinatus traction verification

**Figure 3:**
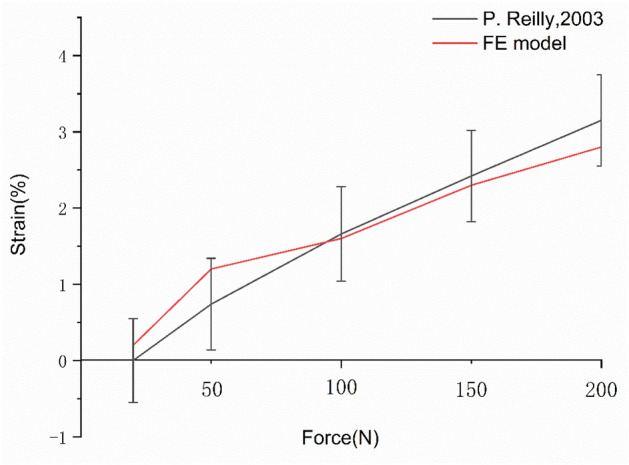
Validation results of supraspinatus tendon strains. Strain of the supraspinatus tendon at the articular side under tensile load from 20 to 200 N at 0˚ of abduction.

The strain during the supraspinatus tensile test was simulated and compared with the results in the literature, which were all within the standard deviation range of the experiment, as shown in Fig. 3.

### Effect of different loads on shoulder joint tissue

#### Risk of supraspinatus muscle injury

**Figure 4:**
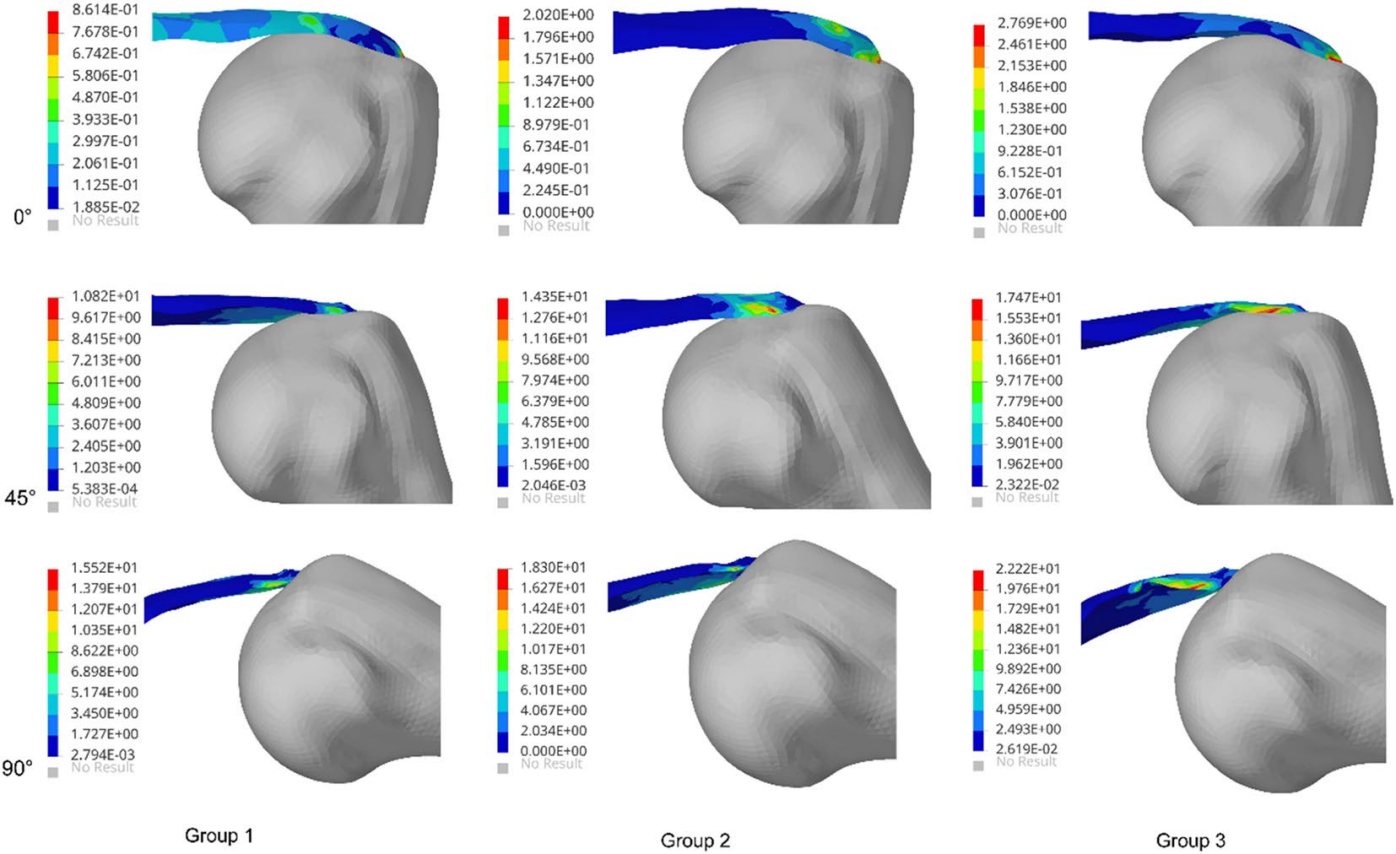
Stress of the supraspinatus in the three groups.

**Figure 5:**
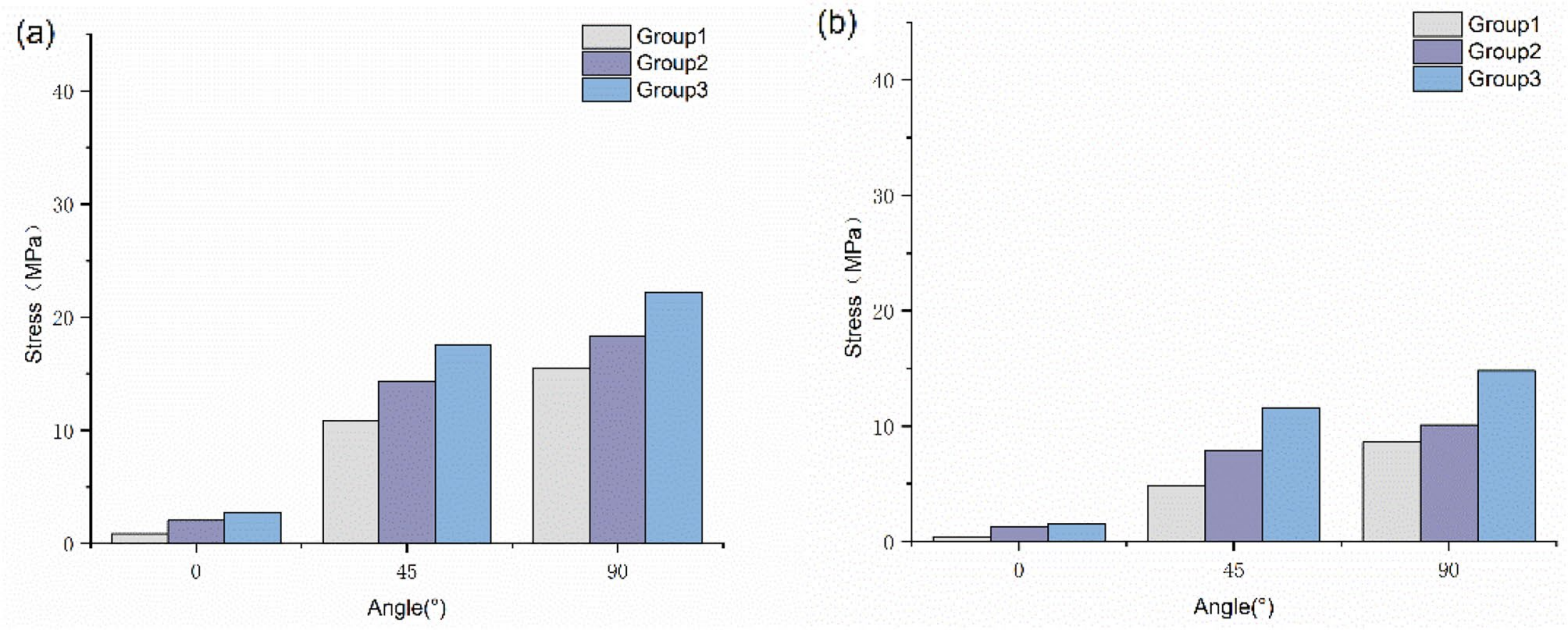
Stress of the articular side and bursal side in the supraspinatus. (**a**) Stress of the articular side. (**b**) Stress of the bursal side.

The risk of supraspinatus tendon injury is associated with maximum von Mises stress. Figures 4 and 5 show that tendon stress increased with increasing abduction angle, and there was a significant difference in stress between the articular side and the bursa side. At 0° abduction, the difference in stress between the articular side and the bursa side in Group 3 was larger than in the other two groups. With an increasing angle, the stress on both sides increased significantly, but the stress on the articular side increased more obviously. At abduction 90°, the joint stress reached 15.5 MPa, which was 14.7 Mpa higher than that at 0°. With increasing load, the joint stress reached 22.2 Mpa, which was 43% higher than that without load.

##### Quantitative analysis

As shown in the Figs. 4 and 5, the supraspinatus muscle showed its maximum stress at 90°. Based on the previous literature^26^, the stress value of 90° under different loads was selected for quantitative analysis. According to the stress field distribution of the supraspinatus tendon, the stress variance, mean value and dispersion coefficient were analyzed and counted. As shown in Table 2, with increasing angle or increasing load, the peak stress on the supraspinatus muscle increased significantly. These data show that the discrete stress coefficients of Groups 2 and 3 are significantly higher than those of Group 1.

**Table 2:** Element stress field of supraspinatus (MPa).

Group	Minimum	Maximum	Median	Mean	Deviation	Extreme difference	Dispersion coefficient
Group 1	0.00	15.5	0.52	0.76	0.59	15.5	0.77
Group 2	0.00	18.3	0.64	0.82	1.26	18.3	1.22
Group 3	0.00	22.2	0.73	0.97	1.85	22.2	1.91

#### Stress on the anterior, middle and posterior deltoid muscles

**Figure 6:**
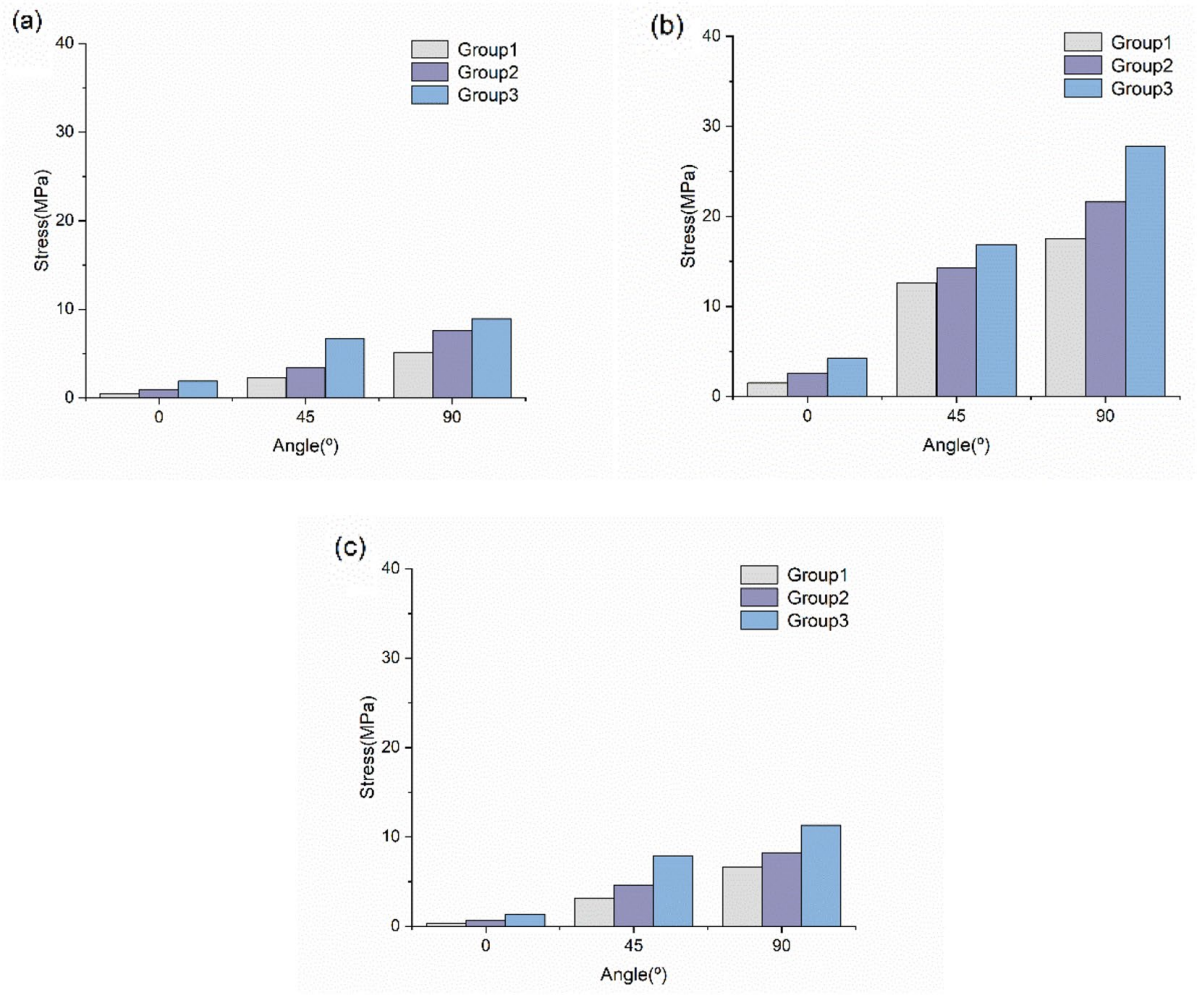
Stress of the anterior, middle and posterior deltoid. (**a**) The maximum von Mises stress of the anterior deltoid. (**b**) The maximum von Mises stress of the middle deltoid. (**c**) The maximum von Mises stress of the posterior deltoid.

Figure 6 shows that the stress on the anterior, middle and posterior deltoid increased to different degrees with increasing abduction angle, especially in the middle deltoid. The stress on the middle deltoid was 1.5 MPa at 0° and it increased 160% in Group 3, while the maximum stress was 27.8 MPa at abduction 90°. The stress of the posterior deltoid was 0.2 MPa at 0°, and that of the anterior deltoid was 0.4 MPa. However, when the third group was abducted at 90°, the stress on the posterior deltoid was 11.3 MPa, which was lower than that of the middle deltoid but 27% higher than that of the anterior deltoid.

#### Strain of the glenohumeral ligament

**Figure 7:**
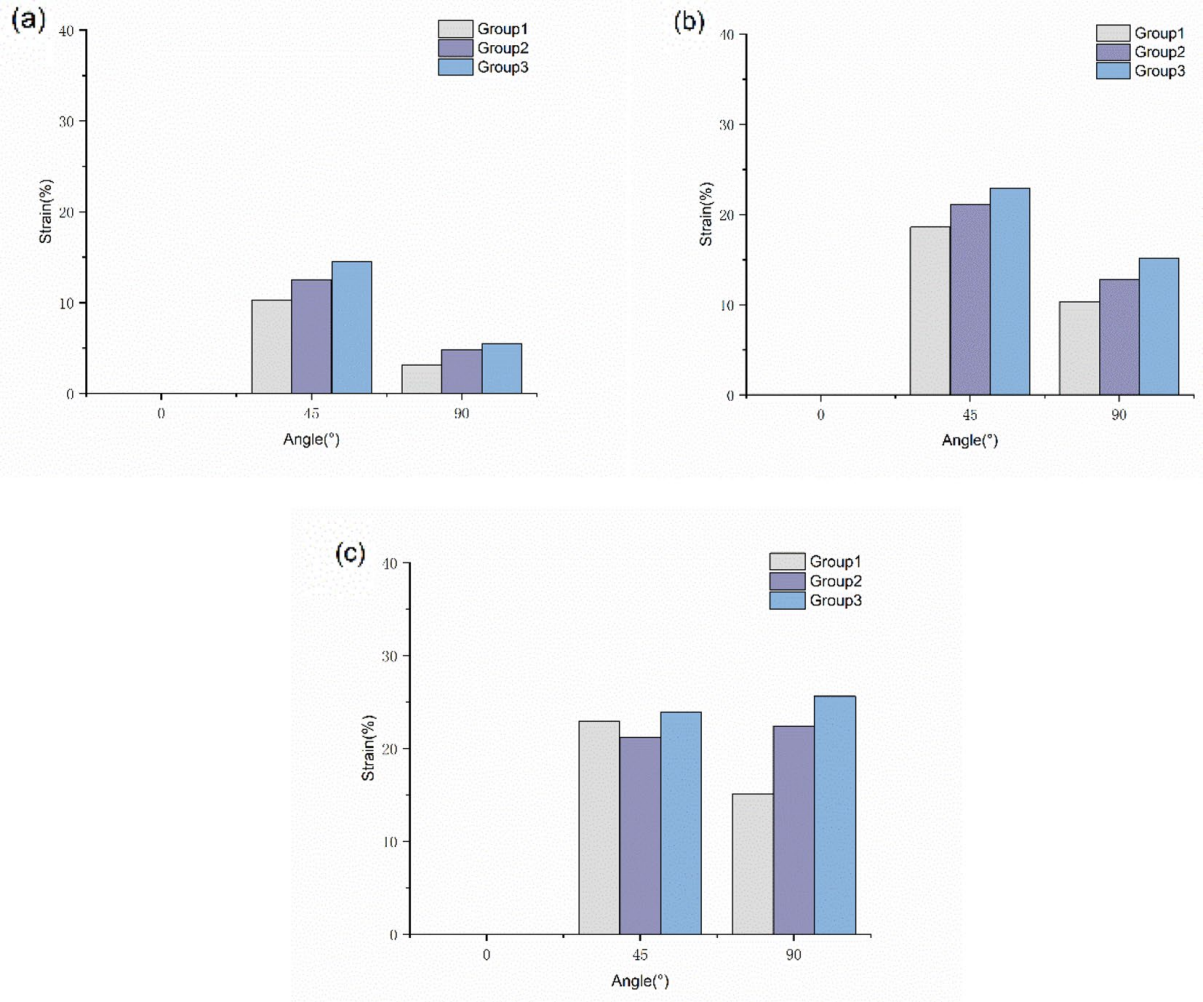
Strain of the glenohumeral ligaments. (**a**) Strain of the superior glenohumeral ligament. (**b**) Strain of the middle glenohumeral ligament. (**c**) Strain of the inferior glenohumeral ligament.

By comparing the initial length of the glenohumeral ligament, the changes in the traction of the glenohumeral ligament during abduction were analyzed to explore the stability of the glenohumeral ligament during abduction. Figure 7 shows that the glenohumeral ligament showed different degrees of strain changes with increasing abduction angle. The strain on the superior and middle glenohumeral ligaments between 0° and 45° is more obvious than that of the inferior and glenohumeral ligaments. However, the strain on the inferior glenohumeral ligament increased significantly after abduction 45°, while the superior glenohumeral ligament showed a downward trend.

## Discussion

Since the shoulder is composed of the humerus, clavicle, scapula, joint capsule and shoulder ligament, the joint support is unstable, thus complicating the finite element analysis for 3D construction of the structure. Because the previous shoulder joint model was not constructed the whole tissue^19^, including the shoulder capsule, bone, ligaments and deltoid muscle. Therefore, a more realistic finite element model of the shoulder joint was constructed by verifying the anatomy data and the supraspinatus muscle in a traction test. Then, we used the model to analyze the effect of different loads on the abductor muscle and the stability of the shoulder joint.

Upper limb load-bearing training is beneficial in daily life and for functional exercise^1,27^. However, load-bearing extravasation causes stress on shoulder tissues and may cause shoulder tissue damage. Through biomechanical analysis of the change in glenohumeral joint translation caused by load bearing abduction, Eleonora Croci et al.^28^ found that load bearing increased the risk of rotator cuff tissue injury. The most common type of rotator cuff tear is a supraspinatus tendon injury, which usually occurs in the critical zone, where the tendon, synovial membrane, and bone tissue are connected, resulting in a concentration of stress and making recovery difficult because of the relatively low blood supply^29^. In the present study, stress changes in the supraspinatus tendon during abduction were evaluated quantitatively. The results showed that the peak stress difference between the articular side and the bursal side of the supraspinatus tendon significantly increased with increasing load during load-bearing abduction, making the stress distribution more uneven and increasing the risk of supraspinatus tendon injury to some extent. Clinical studies^23,30^ found that the stress difference between the articular side and the bursal side of the supraspinatus tendon can cause injury to the supraspinatus tendon. Our results further indicate that the load increases the stress difference between the articular side and the capsular side on the supraspinatus tendon, which increases the risk of injury.

The deltoid muscle provides dynamic stability during different abductive movements of the shoulder. Previous studies found^19,31^ that the movement of upper limbs significantly increases the strain on the middle deltoid muscle but did not analyze the difference in the role of the anterior and posterior deltoid muscles. In our model, the stress on the deltoid muscle increases with increasing abduction, and the stress of the middle deltoid muscle is larger than that of the other muscles, but the stress of the anterior and posterior deltoid muscles also increases to varying degrees. Further comparison showed that the stress on the posterior deltoid muscle is higher than that on the anterior deltoid muscle. This indicates that the anterior and posterior deltoid muscles also provide stability during abduction, not just the middle deltoid muscle. The higher stress of the posterior deltoid muscle may be due to the increase in load changed the motion trajectory, which caused an increase in stress. In addition, the glenohumeral ligament is a statically stable structure of the shoulder joint and is one of the main stabilizing factors of the shoulder during abduction^32^. The finite element model showed that the tensile strain of IGHL is the most obvious with increasing abrasion and load, which is more likely to cause tissue damage. The IGHL is an important tissue that maintains the anterior stability of the shoulder joint^33^, and an increase in load leads to an increase in its mechanical index, thus affecting the stability of the shoulder joint.

Based on the few studies on the biomechanical environmental changes of load on the whole deltoid muscle, supraspinatus muscle and glenohumeral ligament, we analyze the mechanical differences between the dynamic and static stable structures of the shoulder joint during glenohumeral joint abduction to interpret the effect of load changes on shoulder tissues from multiple perspectives. The increase in stress and strain at these specific sites helps to explain the effects of the loading mechanics and location of the clinical injury on the shoulder tissue.

However, there are some limitations to this study. First, the rotator cuff and deltoid muscle were considered isotropic materials for nonlinear analysis, and scapular activity was not considered. Second, the finite element shoulder joint model was established according to the geometric information from a single subject, and the morphological differences between individuals may lead to differences in the stress position and movement mode. Third, the material parameters of the shoulder joint are different from those of the real human body, and there is a certain error, which needs to be improved. Fourth, finite element analysis cannot completely simulate the internal environment of the human body, and its dynamic biomechanical properties still need further experimental analysis.

## Conclusion

In this study, a nonlinear shoulder joint model was established and applied to analyze the stress and strain of the surrounding tissues during glenohumeral joint abduction under load-bearing conditions to provide a mechanical basis for clinical rehabilitation and daily exercise. The results showed that the load increased the stress difference between the articular side of the supraspinatus tendon and the capsular side and led to an increase in the mechanical indices of the middle and posterior deltoid muscle, as well as the inferior glenohumeral ligament. The increased stress and strain in these specific sites increased the risk of tissue injury and affected the stability of the shoulder joint.

## Data Availability

The datasets analyzed during the current study are available from the corresponding author on reasonable request.
